# Impairments in Goal-Directed Actions Predict Treatment Response to Cognitive-Behavioral Therapy in Social Anxiety Disorder

**DOI:** 10.1371/journal.pone.0094778

**Published:** 2014-04-11

**Authors:** Gail A. Alvares, Bernard W. Balleine, Adam J. Guastella

**Affiliations:** Brain & Mind Research Institute, The University of Sydney, Sydney, New South Wales, Australia; University of Tasmania, Australia

## Abstract

Social anxiety disorder is characterized by excessive fear and habitual avoidance of social situations. Decision-making models suggest that patients with anxiety disorders may fail to exhibit goal-directed control over actions. We therefore investigated whether such biases may also be associated with social anxiety and to examine the relationship between such behavior with outcomes from cognitive-behavioral therapy. Patients diagnosed with social anxiety and controls completed an instrumental learning task in which two actions were performed to earn food outcomes. After outcome devaluation, where one outcome was consumed to satiety, participants were re-tested in extinction. Results indicated that, as expected, controls were goal-directed, selectively reducing responding on the action that previously delivered the devalued outcome. Patients with social anxiety, however, exhibited no difference in responding on either action. This loss of a devaluation effect was associated with greater symptom severity and poorer response to therapy. These findings indicate that variations in goal-directed control in social anxiety may represent both a behavioral endophenotype and may be used to predict individuals who will respond to learning-based therapies.

## Introduction

The diagnostic hallmarks of social anxiety disorder include debilitating fears of negative evaluation and avoidance of associated social situations. These persistent and repetitive expectations of negative judgment occur independently to the actual probability of such outcomes occurring [1]–[3]. Such beliefs and behaviors are insensitive to actual experienced outcomes, which may indicate a loss of volitional control. Recent reviews have highlighted how anxious or stressed states can result in decision-making deficits, with a shift from volitional, goal-directed control to inflexible habitual responses [4], [5]. However, there has been little empirical investigation into how such processes contribute to the etiology or maintenance of anxiety disorders, particularly social anxiety. Of further importance for such research is the extent to which biases in decision-making may predict the success of learning-based psychological treatment.

Models of action control highlight the importance of a dual-process that mediates value-based decision-making behavior: (i) a goal-directed system, in which responses are performed with respect to the value of subsequent outcomes (R-O), and actions that are (ii) habitual, controlled by antecedent stimulus-response associations (S-R) [6]–[9]. The ability to flexibly shift between these forms of action control is both necessary and adaptive. Although habitual actions can be executed quickly with relatively low cognitive load, they are relatively inflexible and insensitive to changes in outcome value; goal-directed actions, however, require more time to execute, but are volitional and flexibly adapt to changes in outcome value. These systems co-exist and compete for behavioral control in adaptive decision-making. Critically, if the integrity of the neural systems mediating the goal-directed system is compromised, volitional behavior is impaired and actions become divorced from the value of their respective outcomes.

A key assessment of identifying the current form of action control is outcome devaluation. This involves changing the expected value of a food outcome [10], with the most common method employing sensory specific satiety [11]–[13] and subsequently assessing changes in action performance. If such devaluation produces a change in the performance of actions that previously delivered the devalued outcome, relative to actions that delivered still-valuable outcomes, then behavior is said to be goal-directed. Alternatively, maintained responding for both valued and devalued outcomes following devaluation indicates that an action is habitual, or under S-R control, and is not guided by its consequences (reviewed in [6]).

Evidence from healthy individuals suggests that the induction of acute social stress impairs goal-directed actions without impaired causal outcome knowledge [14], [15]. Clinical evidence also suggests that obsessive-compulsive disorder (OCD), previously classed as an anxiety disorder, is associated with loss of action control (‘slips of action’) and impaired knowledge of action-outcome contingencies [13]. However, direct tests of the ability of anxious patients to update outcome values on the basis of experience, and use those values to guide subsequent actions, has not been performed. Thus, the extent to which persistent negative expectations in social anxiety reflects an inability to update value judgments to guide future decisions remains unknown. This loss of goal-directed control over actions, in turn, may be reflected in the extent of an individual’s symptoms, although this has also yet to be investigated.

Of clinical interest is the potential relationship between changes in goal-directed action control and treatment outcome in social anxiety. The current gold-standard treatment for social anxiety disorder is cognitive-behavioral therapy. Whilst effective, reports of only 50% experiencing remission after treatment are common [16]. This failure to respond to treatment may be associated with an inability to update the value of their expectations, whether through inhibitory learning during exposure sessions [17], [18] or through testing beliefs through cognitive therapy [19]. Thus, impairments in flexible, goal-directed control may not only relate to current levels of anxiety symptoms, but limit an individuals’ ability to effectively integrate newly learned information from treatment to then update subsequent actions and thoughts.

The first aim of this study was to examine whether patients diagnosed with social anxiety disorder would show impairments in flexible goal-directed control over actions. We hypothesized that patients with social anxiety would fail to flexibly update their actions following outcome devaluation in a free-response instrumental learning task, indicative of a loss of goal-directed control. Secondly, we predicted that this impairment would be related to current symptom severity. Lastly, we hypothesized that impaired goal-directed control would be associated with poorer treatment outcomes following cognitive-behavioral therapy.

## Materials and Methods

### Participants

Individuals with a primary diagnosis of social anxiety disorder (n = 23) were recruited from the Anxiety Clinic at the Brain & Mind Research Institute, referred for a group-based cognitive-behavioral therapy program. Exclusion criteria included a primary diagnosis of a psychotic or eating disorder, current suicidal ideation, or comorbid Axis II disorders. Patients were not excluded if they also met criteria for one or more comorbid Axis I disorders (*n* = 20), as is typical of community-based patient samples [20]–[22] and included other anxiety, mood, and/or substance dependence disorders. 35% met criteria for another anxiety disorder, 74% with a mood disorder, and 22% with a substance dependence disorder. 54% were taking between one and three psychotropic medications (combinations of antidepressants, anticonvulsants, antipsychotics, mood stabilizers, and substance withdrawal medication). All medication was stabilized for at least one month prior to assessment. The control group consisted of students recruited from the University of Sydney. Exclusion criteria included diagnosis of any mental disorder, current use of psychotropic medication, or dependence on any recreational substance. Of 29 controls recruited for the present study, five were excluded due to technical problems and one excluded due to a gluten allergy. All participants were required to be free of food allergies or insensitivities. Written consent was obtained and the study was approved by the University of Sydney’s Human Research Ethics Committee (12810).

### Materials

Participants in the social anxiety group were assessed on a separate day by authors GAA, AJG, or a registered psychologist, using the Anxiety Disorder Interview Schedule for Adults [23] and DSM-IV-TR criteria [24]. Controls were assessed using a brief interview, based on the Structured Clinical Interview for DSM-IV-TR [25].

Symptom measures used for both groups included the Social Phobia Anxiety Inventory (SPAI; [26]) and the Depression, Anxiety, and Stress Scale (DASS, three subscales; [27]). A total score for the SPAI was used, totaling both social phobia and agoraphobia items for a total social anxiety severity score. As a measure of impulsivity, we used the Barratt Impulsiveness Scale (BIS; [28]) and the Eating Attitudes Test (EAT; [29]) to assess problematic eating behaviors or attitudes. Additional clinical severity questionnaires obtained from the social anxiety group measured fear and avoidance of social situations using the Liebowitz Social Anxiety Scale (LSAS; [30]), Social Interaction Anxiety Scale, and Social Phobia Scale (SIAS and SPS; [31]). General trait anxiety was assessed using the Trait subscale of the State-Trait Anxiety Inventory (STAI; [32]), psychological distress with the Kessler psychological distress scale (K10; [33]), and the Life Interference Scale to assess the extent to which social anxiety affected various components of the participants’ life (LIS; [34]).

**Figure 1:**
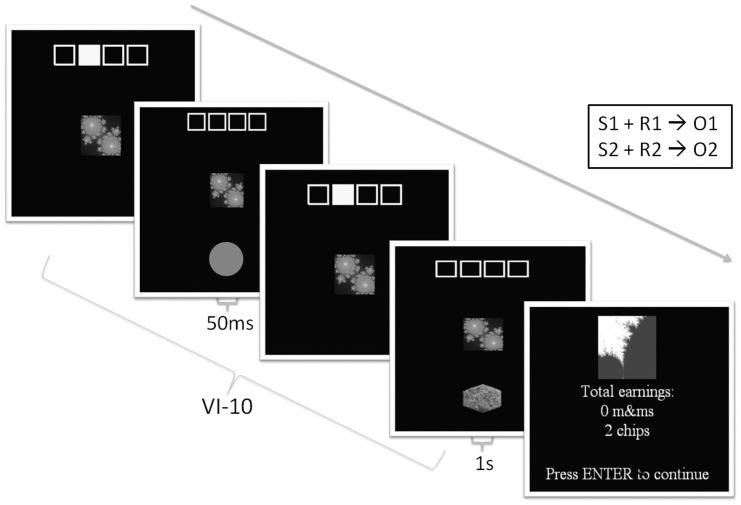
Instrumental task during training. One of two fractal cues was associated with a keyboard button (depicted in the yellow squares) and a food outcome (savory biscuits or chocolate). Food outcomes were available on a variable interval 10 (VI-10) schedule. Trials were presented randomly, lasting between 20 and 40 seconds each within a six minute block. Rest trials in which no reward is available were randomly presented in between trials, lasting for 10 seconds. Cumulative earnings were displayed at the end of each block.

Stimulus presentation and data acquisition for the behavioral task was implemented in Presentation (Neurobehavioral Systems). The task design was based upon a previously published study [11]. In training, individual stimuli (fractal images) were presented and associated with one of two pictures of food outcomes (savory biscuits or chocolate); see Figure 1. During the task, four yellow squares on the screen were presented, corresponding to four yellow keys on the keyboard. Within trials, a fractal cue appeared beneath the highlighted squares. Participants then made actions using the keyboard when the fractal cue appeared, signaling reward availability. As this was a free-response design, analogous to animal paradigms, participants were instructed to respond as much as they preferred on either key. After every action, either a grey circle appeared for 50 ms (indicating no reward), or a picture of a food outcome for 1 second (savory biscuit or chocolate); presentation of a food outcome picture was paired with a sound (bell or click). The picture of the corresponding food outcome was available on a variable-interval 10 schedule (meaning that, on average, one outcome was available every ten seconds upon each key press). In each block of training, there were twelve sets of trials, lasting between 20 and 40 seconds each, with 10 second rest breaks between every one to three trials. After each block, a screen was presented to indicate total earnings of each food outcome, although no actual food was presented during training. The first four controls and five social anxiety participants who completed the task received two blocks of training, lasting six minutes each. Subsequent participants received four blocks of training, lasting six minutes each (while all participants were included in the final analysis, interpretation of findings did not alter when participants who received a shorter amount of training were excluded).

The outcome devaluation procedure involved participants eating one of the food outcomes, counterbalanced across participants, to satiety or until it was no longer pleasant to consume any more [11]. After devaluation, participants completed Likert ratings on contingency awareness (“when this fractal was shown, how likely was it that a button press would result in a chip or m&m reward?”) using a 10-point scale (−5 to 5, ‘m&m more likely’ to ‘chip more likely’) to ensure appropriate acquisition of S-R-O associations. During the extinction test, presentation of the fractal stimuli remained the same as in training, but pictures of outcomes were no longer available after key presses. However, participants were not informed that rewards were no longer available to initially maintain responding rates. Instead, participants were told that the task remained the same, and they could respond as much as they wanted on either key. As in training, counterbalanced trials of 20–40 seconds were presented, with rest periods in-between, for 180 seconds in total. Likert ratings (on a scale of 1–10) for hunger and pleasantness of the two food outcomes were obtained prior to training, post training, and post devaluation.

### Procedure

All participants were instructed to abstain from food and drink (other than water) for two hours prior to the experiment, and from caffeine, nicotine, and recreational substances, on the day. After a complete description of the study, written informed consent was obtained from all participants. After the initial screening interview, all participants completed the common self-report measures and behavioral task. Participants with social anxiety completed the additional questionnaires on a separate day as part of their participation in therapy. Treatment consisted of eight to ten consecutive weeks of group-based cognitive-behavioral therapy lasting between two and three hours per session. After completion of the group, social anxiety participants completed questionnaires a second time as a measure of treatment response.

### Data Analyses

Statistical analyses were performed in SPSS, version 20. Missing values within questionnaires (<20% of individual questionnaires) were replaced with the individual’s mean for that scale or subscale, an imputation strategy valid for smaller percentages of missing data [35]. Missing questionnaires prior to treatment, or questionnaires with more than 20% of missing values, were excluded listwise from analyses. For treatment response, symptom scores for social anxiety participants who did not complete treatment were replaced using the last-observation-carry-forward procedure. Partial η^2^ was used for effect size estimates.

Rates of responding, measured by key presses per second during reward availability for outcomes (over the course of training and during a comparable period in the extinction) were entered into a mixed-design ANOVA, with Devaluation (Valued, Devalued) as the within-subjects variable and Group (Social anxiety disorder, Control) as the between-subjects variable. A suppression ratio was calculated to measure the relative strength of each action during extinction, calculated as the rate of each action (separately for the valued and devalued actions) in extinction, divided by the rate of each action in extinction plus rate during the last block of training. A value between 0 and 0.5 indicates a suppression of responding on that action, whereas a value between 0.5 and 1.0 indicates an elevation in responding. A devaluation ratio was also calculated to measure the relative strength of responding on each action. This was calculated as the rate of responding on the valued action, divided by overall responding on both actions in extinction. Higher values represent more goal-directed actions (a preference for the valued action), and lower values indicate more habitual responding (similar rates of responding on both actions).

Response to treatment was calculated using change scores on self-report measures between the pre and post group research assessments. Associations between symptom severity, treatment response, and behavioral measures were tested using Pearson correlations, two-tailed, with correlation coefficients interpreted as *r* = .5 large, *r* = .3 medium, and *r* = .10 small [36]. Hierarchical multiple regressions were conducted to follow-up any significant correlations. Significance was set at *p*<.05.

Analyses examined (a) whether goal-directed actions in the social anxiety group were impaired, relative to controls; (b) associations between actions and symptom measures; and (c) relationships to treatment outcome.

## Results

### Participant Characteristics

Table 1 summarizes demographic and clinical characteristics between the groups. Groups significantly differed in age and gender distribution (*p*<.05), with the social anxiety group also tending to exhibit greater years of education (*t*(34.08) = 1.85, *p* = .07). As expected, the groups also significantly differed on all self-report measures of social anxiety, mood, and stress (all *p*<.05). However, no differences were observed between groups in body mass index (BMI), self-reported impulsiveness, or problematic eating attitudes, all *p*-values >.05.

**Table 1:** Participant Characteristics.

Variable	SAD	Control	*p*
	*n* = 23	*n* = 23	
Age	25.39 (6.70)	20.70 (5.36)	.01
Gender (M/F)	18/5	11/12	.03
Years of education	12.96 (1.58)	12.26 (0.86)	.07
BMI^a^	23.78 (3.76)	23.12 (2.46)	.50
Smoke (yes/no)	2/21	3/20	.64
SPAI	51.52 (15.02)	19.83 (9.03)	<.001
DASS-Depression	19.78 (9.10)	5.65 (4.54)	<.001
DASS-Anxiety	16.96 (9.46)	6.96 (6.63)	<.001
DASS-Stress	19.04 (9.02)	9.39 (6.89)	<.001
BIS^b^	64.71 (11.39)	64.68 (9.41)	.99
EAT^c^	5.91 (7.13)	6.32 (6.12)	.84

*Note*. *p*-values come from independent samples *t*-tests or chi-square analyses. BMI =  Body Mass Index; SPAI =  Social Phobia Anxiety Inventory; DASS =  Depression, Anxiety, Stress Scale; BIS =  Barratt Impulsiveness Scale, total impulsiveness score; EAT =  Eating Attitudes Test.

a SAD *n* = 21.

b SAD *n* = 21, Control *n* = 22;

c SAD *n* = 22, Control *n* = 22.

### Behavioral Results

**Figure 2:**
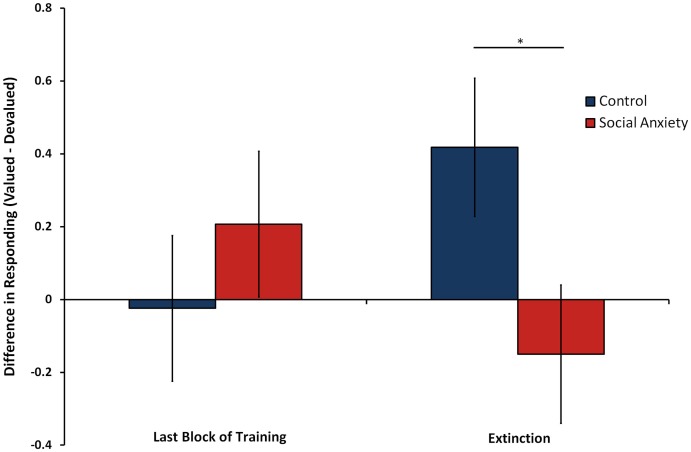
Difference in mean rates of responding for actions predicting valued relative to devalued outcomes over the last block of training and extinction trials in controls compared to social anxiety disorder patients. Error bars depict standard error of the mean difference between controls and patients. *p<.05.

At the last block of training, there were no significant differences between either action for the two food outcomes (*F*(1, 44) = 0.42, *p* = .52, η^2^ = .01), or differences between groups in overall rates of responding (*F*(1, 44) = 0.17, *p* = .68, η^2^ = .004). After devaluation, a significant interaction emerged in the extinction test between group and devaluation in rates of responding (*F*(1, 44) = 4.49, *p* = .04, η^2^ = .09). Pairwise comparisons confirmed that controls exhibited a significant devaluation effect, selectively reducing a devalued action compared to a still-valuable action, indicative of goal-directed responding (*p* = .03). Participants with social anxiety, however, exhibited no effect of devaluation, with no difference in actions made on either key predicting the different outcomes (*p* = .43); see Figure 2. This bias was not due to differences in overall rates of responding between groups during extinction (*F*(1, 44) = 1.00, *p* = .32, η^2^ = .02) or differences in amount of food consumed (*F*(1, 42) = 0.81, *p* = .37, η^2^ = .02; *M* = 61.54 grams, *SD* = 52.74); amount of food consumed was missing for two participants. Interpretation of these results also did not vary when calculating responding as a ratio to the rate of responding at the last block of training, or after adding age, gender, and years of education as covariates (*F*(1, 41) = 4.69, *p* = .04, η^2^ = .10).

To ensure that this lack of devaluation effect was not due to hunger or inadequate knowledge about the change in outcome value after devaluation, analysis was conducted on Likert ratings collected throughout the task. As expected, ratings of hunger significantly decreased for both groups after devaluation (*F*(2, 80) = 49.53, *p*<.001, η^2^ = .55), but did not differ between groups (*F*(1, 40) = 0.12, *p* = .73, η^2^ = .003). Ratings of pleasantness decreased for the devalued outcome after selective satiation for both groups, but not for the valued outcome (*F*(2, 88) = 8.05, *p* = .001, η^2^ = .16), with no differences between groups (*p*>.05). This indicated that whilst both groups retained explicit knowledge about the change in outcome value, the social anxiety group failed to integrate this information to modulate subsequent responding for outcomes during extinction.

### Correlations with Symptom Measures

Using an overall devaluation ratio to measure relative goal-directed or habitual behavior, and suppression ratios for valued and devalued actions, bivariate Pearson correlations were run to examine relationships between these measures and clinical self-reports within the social anxiety group. Consistent with the original Barratt theory of impulsiveness [28], the devaluation ratio was significantly negatively correlated with overall impulsivity (*r* = −0.46, *p* = .04). This indicated that increasing levels of impulsivity in the social anxiety group was associated with a significantly poorer ability to reduce responding on the devalued action. In terms of suppression ratios, responding on the valued action during the extinction phase was also associated with increased impulsivity (*r* = −0.47, *p* = .03), overall trait anxiety (STAI *r* = −0.55, *p* = .01), psychological distress (K10; *r* = −0.45, *p* = .04), and greater life interference (LIS; *r* = −0.53, *p* = .01). No significant correlations were observed for suppressed responding on the devalued action.

### Response to Treatment

In terms of treatment outcome, 73.91% of the patients with social anxiety completed the group cognitive-behavioral therapy program. Significant positive correlations emerged between participants’ devaluation ratio prior to entering treatment and change scores on the LSAS (*r* = 0.43, *p* = .04) and LIS (*r* = 0.49, *p* = .02), with borderline trends exhibited for the stress subscale of the DASS (*r* = 0.41, *p* = .05), STAI (*r* = 0.41, *p* = .05), and SPS (*r* = 0.37, *p* = .08). This indicated that changes in action control prior to treatment were related to treatment response.

Hierarchical multiple regressions were then conducted on these outcomes, with age, gender and baseline severity for each outcome entered as initial predictors of changes in symptom severity, with the devaluation ratio entered as a second step. Results indicated that, even after accounting for these other predictors, action control predicted changes in treatment outcome across social anxiety symptoms, life interference, and general trait anxiety; see Table 2. That is, reductions in goal-directed actions were related to poorer outcomes after treatment, whereas those patients with less impaired goal-directed responding prior to treatment later showed greater reductions in social anxiety symptoms.

**Table 2:** Hierarchical Multiple Regression Analyses Predicting Treatment Outcome from Goal-Directed Actions at Prior to Treatment.

	Change from Baseline to Post-Treatment														
	LSAS			LIS			DASS-Stress			STAI-Trait			SPS		
Predictor	B	SE B	β	B	SE B	β	B	SE B	β	B	SE B	β	B	SE B	β
Step 1															
Constant	16.96	14.44		13.69	7.17		11.86	7.12		5.55	9.91		11.81	7.24	
Age	−0.40	0.43	−.28	−0.44	0.20	−.61	−0.41	0.19	−.49	−0.44	0.20	−.51*	−0.40	0.27	−.39
Gender	−6.4	7.31	−.28	−3.70	2.87	−.32	−1.51	3.43	−.11	−5.30	3.26	−.38	−5.12	4.42	−.31
Baseline Symptoms^a^	0.03	0.11	.08	0.15	0.16	.24	0.15	0.14	.25	0.25	0.14	.37	0.09	0.09	.24
R^2^			.07			.24			.23			.29			.13
Step 2															
Constant	−29.88	20.26		−6.66	10.42		3.24	13.12		−16.42	12.53		−31.10	12.17	
Age	−0.40	0.39	−.29	−0.44	0.17	−.62*	−0.38	0.20	−.46	−0.41	0.18	−.48*	−0.44	0.20	−.43*
Gender	−8.0	6.64	−.38	−4.40	2.52	−.38	−1.40	3.47	−.10	−5.88	2.88	−.42	−7.77	3.32	−.47*
Baseline Symptoms^a^	0.06	0.10	.15	0.19	0.14	.31	0.13	0.15	.21	0.23	0.13	.35	0.17	0.07	.47*
Devaluation Ratio	79.39	35.35	.48 *	40.11	16.41	.48*	16.73	21.31	.17	45.67	18.71	.45 *	83.82	21.36	.70***
R^2^			.29			.45			.26			.48			.56
ΔR^2^			.22			.22			.03			.19			.43

*Note*. *p<.05 ***p≤.001. LSAS =  Liebowitz Social Anxiety Scale; LIS =  Life Interference Scale; DASS = Depression, Anxiety, Stress Scale; STAI =  State-Trait Anxiety Inventory; SPS =  Social Phobia Scale.

a =  baseline scores for each outcome.

## Discussion

The results from the present study demonstrated that individuals diagnosed with social anxiety disorder exhibited impairments in flexible goal-directed actions. Patients failed to use information about explicit changes in outcome value to flexibly guide their subsequent actions, compared to controls. This behavioral bias was associated with increased impulsivity, general anxiety, and psychological distress and was not due to inadequate knowledge about the change in outcome value. When the social anxiety group was subsequently assessed at the completion of cognitive-behavioral therapy, impaired goal-directed actions predicted poorer response to treatment. The results, therefore, suggest that a loss of goal-directed control over actions may contribute to the maintenance of social anxiety symptoms in social anxiety disorder, and assist with predicting individuals that do not respond to treatment.

The initial finding that social anxiety disorder is associated with a loss of goal-directed action control is consistent with previous studies demonstrating effects of both acute [14] and chronic [37] stress using similar tasks, albeit in normal adults and rodent models. However, the finding that reduced performance in extinction for actions that earned valuable outcomes was associated with greater anxiety and psychological distress is of particular interest. Recent reviews have argued that changes in emotional states, due to deficits in top-down mechanisms required for appropriate action control, could mediate both the behavioral inflexibility and negatively biased cognition associated with anxiety [4], [38]. Such arguments are consistent with approach-avoidance models [39], that suggest that anxiety or stress is associated with an inhibition of approach towards potentially rewarding outcomes, and a bias towards potentially aversive consequences. As anxiety has been argued to be related to an inability to process rewarding information appropriately to guide actions [4], social anxiety disorder may therefore reflect a dysregulation in the ability to adequately integrate knowledge about changes in outcome value to subsequently guide approach-related actions, independent of any social stimuli or stress induction. This may be particularly pronounced in those patients exhibiting higher levels of psychological distress and anxiety.

In terms of treatment response, individuals with social anxiety disorder exhibiting less goal-directed actions predicted poorer response to gold-standard cognitive-behavioral therapy. Such treatments aim to enhance approach with feared social stimuli, whilst reducing negative beliefs about expected outcomes. Specifically, learning that a feared outcome will not occur during exposure therapy is considered the analog to extinction in animal learning models. Although this study cannot determine how causal the relationship between goal-directed behavior and treatment response is, it does suggest that resistance to extinction during therapy is partially due to an inability to integrate changes in information from the environment to guide subsequent decision-making. Thus, individuals who are better at updating their actions with respect to changes in an expected outcome in the environment may be more likely to respond best to such treatment.

A range of hypotheses have been put forward to explain why stress and anxiety biases the expression of habitual actions, including frontostriatal reorganization [37], striatal dysfunction [40], noradrenergic activity [41], or possibly involvement with the autonomic nervous system [40]. In healthy humans, habitual actions are associated with increased activity in the posterior putamen [11] and stronger white matter tract connectivity between the putamen and premotor cortex [42], whilst goal-directed behaviors activate prefrontal regions [12], [43] and are associated with increased white matter connectivity between the ventromedial prefrontal cortex and the caudate [42]. These dissociable pathways are likely the human homologs of well-established regions in rodent models, specifically the dorsolateral and dorsomedial striatum [6]. Limited volumetric neuroimaging studies have been conducted in social anxiety, however study some evidence suggests that there is a greater age-related changes in putamen volume in patients with social anxiety, compared to controls [44]. Together these studies suggest that reward circuitry, specifically the basal ganglia and its connections to frontal and motor regions, may play a role in biasing reward-guided actions in social anxiety disorder. It may be predicted, for example, that patients with social anxiety with a greater bias in impaired goal-directed actions will be reflected in stronger white matter connections between putamen and premotor cortex, or enhanced functional activation of the putamen during extinction.

Although there were significant baseline differences in age and gender distribution between groups, and more significant medication use and comorbidity within the social anxiety group, these differences were not expected to modify behavior in this type of task. Indeed, covarying for age, gender, and years of education, did not change interpretation of the present findings. Additionally, psychotropic medication used by patients in this study were all stabilized for at least four weeks prior to testing and the extent of comorbidities were consistent with our use of typical community samples of social anxiety disorder patients [22]. However, future research could examine anxious patients independent of any psychotropic medications and the potential impact of comorbidity on behavioral responses. We also observed associations between loss of goal-directed control over actions and response to cognitive-behavioral therapy in social anxiety disorder. However, it is not known whether this loss also predicts response to other types of treatments, including other non-learning based therapies or pharmacological interventions. Future research in this area may help to identify the specific mechanism by which individual differences in impaired goal-directed actions predicts response to treatment.

In conclusion, the present study demonstrated that social anxiety disorder is associated with impaired goal-directed control over actions after specific outcome devaluation. This loss was associated with impulsivity, psychological distress, and predicted poorer response to cognitive-behavioral therapy. Such findings indicate that biases in action control may not only represent a behavioral endophenotype but may predict treatment response to learning-based therapies in social anxiety disorder.
